# Physical activity, screen time, and outdoor learning environment practices and policy implementation: a cross sectional study of Texas child care centers

**DOI:** 10.1186/s12889-019-6588-5

**Published:** 2019-03-07

**Authors:** Courtney E. Byrd-Williams, Erin E. Dooley, Christina A. Thi, Cari Browning, Deanna M. Hoelscher

**Affiliations:** 1 Michael & Susan Dell Center for Healthy Living at The University of Texas Health Science Center (UTHealth) School of Public Health in Austin, 1616 Guadalupe, Suite 6.300, Austin, TX 78701 USA; 2 Department of State Health Services, Obesity Prevention Program, Health Promotion and Chronic Disease Prevention Section, MC 1965, PO Box 149347, Austin, TX USA

**Keywords:** Pediatrics, Physical activity, Environment, Guidelines and recommendations, Public health

## Abstract

**Background:**

Early care and education (ECE) centers are important for combating childhood obesity. Understanding policies and practices of ECE centers is necessary for promotion of healthy behaviors. The purpose of this study is to describe self-reported practices, outdoor environment aspects, and center policies for physical activity and screen time in a statewide convenience sample of non-Head Start Texas ECE centers.

**Methods:**

Licensed home and child care centers in Texas with email addresses publicly available on the Department of Family and Protective Services website (*N* = 6568) were invited to participate in an online survey. Descriptive statistics of self-reported practices, policies, and outdoor learning environment are described.

**Results:**

827 surveys were collected (response rate = 12.6%). Exclusion criteria yielded a cross-sectional sample of 481 center-only respondents. > 80% of centers meet best practice recommendations for screen time practices for infants and toddlers, although written policies were low (M = 1.4 policies, SD = 1.65, range = 0–6). For physical activity, < 30% meet best practice recommendations with M = 3.9 policies (SD = 3.0, range = 0–10) policies reported. Outdoor learning environment indicators (M = 5.7 policies, SD = 2.5, range = 0–12) and adequate play settings, storage (< 40%), and greenery (< 20%) were reported.

**Conclusions:**

This statewide convenience sample of non-Head Start Texas ECE centers shows numerous opportunities for improvement in practices and policies surrounding outdoor environments, physical activity, and screen time. With less than half of centers meeting the recommendations for physical activity and outdoor learning environments, dedicating resources to help centers enact and modify written policies and to implement programs to improve their outdoor learning environments could promote physical activity and reduce sedentary time of children.

**Electronic supplementary material:**

The online version of this article (10.1186/s12889-019-6588-5) contains supplementary material, which is available to authorized users.

## Background

Promoting daily physical activity and limiting sedentary time in the preschool years is important for preventing excessive weight gain and childhood obesity [1]. As approximately 70% of children, ages 4–5 years, attend center-based child care programs for an average of 30 h per week [2] in the US, early care and education (ECE) centers are an important setting to shape healthy behaviors. Unfortunately, many children are not meeting daily recommendations for physical activity and are spending a large percent of their time in ECE in sedentary activity [3].

The social ecological model suggests there are individual, social, and physical environment determinants of recreational physical activity [4, 5]. As young children are dependent on their caregivers for opportunities to be active in the ECE setting, social and environmental aspects that promote physical activity are paramount. Social aspects, such as peers to play with [6] and child care provider behavior [7], influence activity. The physical environment, including open, grassy spaces and equipment such as balls or wheeled toys, also increase physical activity in preschool children [6, 8]. Additionally, characteristics of the ECE center’s outdoor learning environment, such as looped pathways, have been found to increase preschoolers’ physical activity [9–11].

Although it has been found that Texas has a higher than average number (7 of 15) of state-level regulations consistent with National Academy of Medicine recommendations for ECE centers in the US [12], the extent to which Texas ECE centers are meeting policy and practice recommendations for physical activity, screen time, and the outdoor learning environment is unknown. Thus, the primary aim of this study is to examine the first statewide, convenience sample in Texas of physical activity- and screen time-related policies, practices, and outdoor characteristics in non-Head Start child care centers. Results from this study can help to identify opportunities for improvement within ECE centers in an effort to increase children’s physical activity.

## Methods

### Study design

The data for this cross-sectional, descriptive analysis was collected as part of the Early Childhood Physical Activity Survey administered by the Texas Department of State Health Services (DSHS) Health Promotion and Chronic Disease Prevention Section (HPCDPS) on behalf of the Early Childhood Health and Nutrition Interagency Council (the Council). The Council was created by Senate Bill 395, during the 81st Regular Session of the Texas Legislature, to improve the health of Texas infants and children under the age of six [13].

In January 2016, a listserv of child care facility email addresses was created from publicly available data on the Department of Family and Protective Services website [14]. There were a total of 7542 public email addresses available out of 15,789 child care facilities in Texas. After removing duplicate emails (e.g. one owner/director of several facilities), the listserv totaled 6561 unique email addresses; an additional 7 email addresses were added at the request of the child care facilities, for a total of 6568 email addresses. The open, online survey was conducted in February 2016, using software available in both English and Spanish. The software did not allow multiple responses from an email address. A total of three reminders were sent to complete the survey. Instructions stated that the survey should be completed by a person responsible for overseeing the physical activity of the children in care (e.g., day care home provider, center teacher, center director, or administrator). Participation was assured to be confidential, and no incentives were offered. The institutional review board at The University of Texas Health Science Center at Houston approved all protocols and procedures. This study complies with the Checklist for Reporting Results of Internet E-Surveys (CHERRIES) [15].

### Participants

**Fig. 1:**
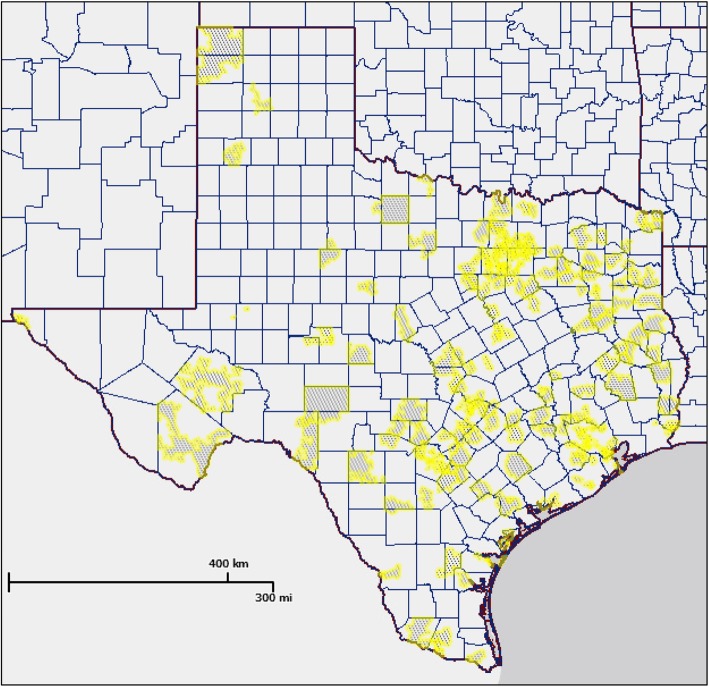
Map of Participating Respondent Distribution for the Early Childhood Physical Activity Survey Map of participating respondent distribution for the Early Childhood Physical Activity Survey. The gray shaded areas with yellow boundaries represent the center zip codes of survey respondents.

A total of 827 complete or partially complete responses were recorded, resulting in a 12.6% participation rate (827/6568). Participants were included in this study if they indicated that: a) they worked in a child care center, b) their current position was center director, center teacher, or administrator, and c) the center enrolled infants, toddlers, and/or preschoolers. Exclusion criteria included: a) employment in a child care home, Head Start, Early Head Start, or state-funded pre-k program (*n* = 212), b) home-based providers or principals (*n* = 20), c) the center only enrolled children over the age of 6 (*n* = 5) or age of children enrolled were missing (*n* = 4), and d) > 60% of survey responses were missing, which was typically after questions regarding center characteristics (*n* = 105). Head Start and Early Head Start programs were excluded from the analysis because they are funded by the U.S. Department of Health & Human Services to promote school readiness of young children from low-income families and have additional or different standards for physical activity and screen time behaviors than non-federally funded child care programs. State-funded pre-k programs were also excluded from analyses for similar reasons. Additionally, child care homes were excluded due to differences in Texas licensing standards for home- and center-based care. In total, 481 participants, each representing a different child care center, were included in the analysis. A map of the respondent distribution can be seen in Fig. 1**.**

### Measures

The Early Childhood Physical Activity Survey (see Additional file 1) was developed using questionnaire items from the Nutrition and Physical Activity Self-Assessment for Child Care assessment tool (NAP SACC) [16, 17], Yale Rudd Center Child Care Nutrition and Physical Activity Assessment [18], Texas Childhood Obesity Research Demonstration (CORD) child care survey [19, 20], the Natural Learning Initiative’s (NLI) Best Practice Indicators for a Model Outdoor Learning Environment Toolkit [21], and other items developed specifically for this study. Longer instruments, such as the Environment and Policy Evaluation and Observation - Self-Report (EPAO-SR) [22], were considered but ultimately the instruments used were chosen to increase responses and allow for comparison with other statewide surveys. The survey was a collaborative effort among the Council members and stakeholders and reviewed by the DSHS physical activity coordinator for face validity prior to dissemination. The final survey contained 38 questions about center characteristics, participant demographics, physical activity and screen time practices, written policies, barriers to promoting physical activity, types of physical activity equipment and resources used outdoors, and physical activity training for staff. DSHS Translation Services translated the Early Care Physical Activity Survey and accompanying emails to Spanish. The survey took approximately 15 min to complete.

There were two required adaptive yes/no questions placed within the survey: “Does your facility have a written policy on physical activity and/or screen time?” and “Teachers and staff received professional development on children’s physical activity.” If participants answered “no” or “never”, respectively, then the subsequent questions pertaining to topics addressed by physical activity and screen time policies and professional development were skipped. The questions about center characteristic and respondent demographics were required. The survey was tested for technical functionality by DSHS HPCDPS and Council members before administration.

### Physical activity and screen time practices

There were 14 questions regarding physical activity and screen time practices. Each physical activity practice question had four unique, practice-specific response options, including the best practice recommendation for that item. Two questions assessing educational and recreational screen time had open-ended responses; numerical responses were summed into a total screen time amount. The continuous variables were transformed into categorical options that aligned with answer choices on the NAP SACC Screen Time assessment [17]. Best practice recommendations for physical activity and screen time were modeled from the recommendations provided by Let’s Move! Child Care [23], SHAPE America (formerly National Association for Sport and Physical Education (NASPE)) [24], American Academy of Pediatrics [25], Caring for our Children [26], and NAP SACC [16, 17].

### Outdoor learning environment practices

There were three questions regarding outdoor learning environments. Two questions had four unique, practice-specific response options. One question, “Which of the following best practice indicators for a model outdoor learning environment does your facility include?” asked respondents to choose all of the 12 key indicators for outdoor learning environments that applied. Best practice recommendations were modeled in accordance with the Best Practice Indicators for a Model Outdoor Learning Environment by the Natural Learning Initiative’s (NLI) Preventing Obesity by Design (POD) [21]. The check all that apply options were dichotomized into reported/not reported.

### Physical activity and screen time policies

There were three questions regarding physical activity and screen time policies. The first question was the adaptive yes/no question. If participants answered “yes” to having a written policy, then two questions assessed physical activity and screen time policies. There were 13 policy options for physical activity and 9 policy options for screen time policies. Policy options were dichotomized into reported/not reported.

### Statistical analysis

Descriptive analyses were conducted on survey responses. We computed means, frequencies, and percentages of responses. All statistical analyses were performed using SAS software version 9.4 (SAS Institute Inc., Cary, NC, USA).

## Results

A total of 481 completed surveys were included in analysis. All surveys were completed in English except for four, which were completed in Spanish. The ECE center and participant descriptive characteristics are listed in Table 1. Most respondents were Non-Hispanic, white, female, spoke mostly English, and had some college, technical degree, or higher.

**Table 1:** Characteristics of Participating Texas Early Childhood Physical Activity Survey Participants (*N* = 481)^a^

	%	(*n*)
Center characteristics		
Enrollment		
Infants (< 12 months)	63.41	(305)
Toddlers (12–23 months)	83.58	(402)
Preschool (2–5 years)	98.75	(475)
Primary language spoken at center		
Only English	42.29	(192)
More English than another	40.09	(182)
Both English and Spanish	16.52	(75)
More Spanish than another	0.66	(3)
Language other than English or Spanish	0.44	(2)
Participant characteristics		
Age (M, SD)	49.30 (10.36)	
Female	97.27	(428)
Highest grade completed		
High school or lower	4.70	(21)
Child Development Associate (CDA)	9.17	(41)
Some college/ technical school	31.10	(139)
College Graduate	38.03	(170)
Graduate Degree	17.00	(76)
Ethnicity		
Hispanic	13.96	(62)
Not Hispanic/ Latino	86.04	(382)
Race		
White	86.14	(379)
Black or African American	8.41	(37)
Asian	0.91	(4)
Other	4.54	(20)

^a^Total *N* = 481. Actual n varies due to missing data

### Physical activity and screen time practices

The reported physical activity and screen time-related practices are shown in Table 2. Overall, a majority of centers self-reported meeting the minimum best practice for providing at least 60 min of indoor and outdoor physical activity for toddlers and preschoolers; however, only a small number have practices in accordance with the NAP SACC recommendations for toddlers (26.57%) and preschoolers (20.72%) of providing over 90 and 120 daily minutes of indoor and outdoor physical activity, respectively. Less than 15% of centers meet the recommendations of 60 min of structured physical activity provided by SHAPE America, American Academy of Pediatrics, and NAP SACC. More than half (56.58%) report meeting the Let’s Move! Child Care and Caring for our Children recommendation for 15 min or less infants spend in confining equipment; however, less than 15% report meeting the NAP SACC best practice recommendation of never placing infants in seats, swings, or ExerSaucers outside of nap and meal times. For toddlers and preschoolers, more than half (52.81%) of participants reported meeting the NAP SACC and Caring for our Children practice recommendation for seated time. For staff specific behaviors, only 30% of centers meet the best practice recommendation from NAP SACC and National Academy of Medicine of having staff join children in physical activity, however 70% indicated that staff do not withhold physical activity as punishment, meeting recommendations from NAP SACC, Caring for our Children, and the National Academy of Medicine.

**Table 2:** Reported Practices Regarding Physical Activity and Screen Time in Participating Texas Child Care Centers

	Expected *n*^a^	% (*n*)	
Infants Only			
Developmentally appropriate play equipment offered during tummy time & indoor activities^b^	305		
Rarely or never		1.17	(4)
Sometimes		3.23	(11)
Often		25.22	(86)
**Always**		**70.38**	**(240)**
Our program offers “tummy time” to non-crawling infants^b^			
1x per day or less (Half-day 1x every other day)		3.06	(10)
2x per day (Half-day 1x per day)		19.88	(65)
3x per day (Half-day 2x per day)		27.22	(89)
**4x per day (Half-day 2x per day)**		**49.85**	**(163)**
Outside of nap/meal times, infants are placed in seats, swings, ExcerSaucers^b^			
30+ min		5.50	(18)
15–29 min		37.92	(124)
1–14 min		43.12	(141)
**Never**		**13.46**	**(44)**
Toddlers Only			
Indoor and outdoor PA provided^b^	402		
< 60 min (Half-day 30)		15.79	(62)
60–74 min (Half-day 15–29)		42.86	(159)
75–89 min (Half-day 30–44 min)		14.79	(55)
**≥ 90 min (Half-day 45+ min)**		**26.57**	**(98)**
Preschoolers Only			
For children ≥2 yrs., the amount of recreational screen time allowed each week^b^	475		
**≥** 90 min		9.31	(39)
60–89 min		6.92	(29)
30–59 min		12.89	(54)
**< 30 min**		**70.88**	**(297)**
For children ≥2 yrs., the amount of educational screen time allowed each week^b^			
**≥** 90 min		11.57	(50)
60–89 min		10.65	(46)
30–59 min		20.37	(88)
**< 30 min**		**57.41**	**(248)**
For children ≥2 yrs., the amount of total screen time allowed each week^b^			
**≥** 90 min		21.22	(94)
60–89 min		14.22	(63)
30–59 min		15.35	(68)
**< 30 min**		**49.21**	**(218)**
Indoor and outdoor PA provided^b^			
< 60 min (Half-day 30)		8.88	(42)
60–89 min (Half-day 30–44)		47.78	(226)
90–119 min (Half-day 45–59 min)		22.62	(107)
**≥ 120 min (Half-day 60+ min)**		**20.72**	**(98)**
Adult-led (structured) PA provided^b^			
< 30 min (Half-day 10)		28.27	(134)
30–44 min (Half-day 10–19)		41.77	(198)
45–59 min (Half-day 20–29 min)		15.19	(72)
**≥ 60 min (Half-day 30+ min)**		**14.77**	**(70)**
Infants and Toddlers			
For children < 2 yrs., the amount of recreational screen time allowed each week^b^	406		
**≥** 60 min		3.35	(12)
30–59 min		1.68	(6)
1–29 min		7.26	(26)
**No screen time**		**87.71**	**(314)**
For children < 2 yrs., the amount of educational screen time allowed each week^b^			
**≥** 60 min		3.77	(14)
30–59 min		4.58	(17)
1–29 min		8.36	(31)
**No screen time**		**83.29**	**(309)**
For children < 2 yrs., the amount of total screen time allowed each week^b^			
**≥** 60 min		5.59	(21)
30–59 min		5.85	(22)
1–29 min		7.18	(27)
**No screen time**		**81.38**	**(306)**
Toddlers and Preschoolers			
Outside of nap & meals, the longest time expected to be seated at one time^b^	479		
**≥** 30 min		1.52	(7)
20–29 min		14.72	(68)
15–19 min		30.95	(143)
**< 15 min**		**52.81**	**(244)**
**All Ages**			
PA education (motor-skill development) provided through standardized curriculum^b^	481		
Never		15.11	(71)
1x per month		1.06	(5)
2-3x per month		7.87	(37)
**1x per week**		**75.96**	**(357)**
Staff members restrict active play time for children who misbehave^c^			
All staff members		3.18	(15)
Most staff members		1.69	(8)
Some staff members		24.58	(116)
**Never**		**70.55**	**(333)**
Teachers/staff receive professional development on children’s PA^b^			
Never		6.77	(31)
< 1x per year		19.43	(89)
1x per year		39.96	(183)
**≥ 2x per year**		**33.84**	**(155)**
During unstructured PA playtime, teachers/ caregivers join children in active play^d^			
Rarely or never (mostly sit or stand)		2.30	(11)
Sometimes join children		38.70	(185)
Often or always join children		28.87	(138)
**Often or always join children & make positive statements**		**30.13**	**(144)**
The facility shows visible PA support (poster, pictures, or books)^d^			
No support items are displayed		12.85	(60)
A few support items is displayed in a few rooms		34.26	(160)
Support items are displayed in most rooms		30.19	(141)
**Support items are displayed in all rooms**		**22.70**	**(106)**

NOTE: Infant (≤12 mos); Toddler (13–23 mos); Preschool (2–5 yrs)

Boldface indicates best practice recommendation

^a^Expected n refers to the total expected response number based on centers that reported enrolled children within the age bracket. Actual n varies due to missing data

^b^Early Childhood PA Survey Question based on NAP SACC

^c^Early Childhood PA Survey Question based on Yale Rudd Center Child Care Nutrition and PA Assessment

^d^Early Childhood PA Survey Question based on TX CORD

Abbreviations: PA, physical activity; min, minutes; x, time

For screen time behaviors, a majority (> 80%) of centers report that they meet the recommendations for not providing screen time to children under the age of two. However, for preschoolers*,* ages 3–5 years, fewer centers meet the recommendations. One half (50.79%) of centers allowed more than 30 min of screen time per week. When examining screen time stratified by educational or recreational minutes provided, 70% of centers provide less than 30 min of recreational screen time per week; however, 42% provide minutes in excess of educational time recommendations (30 min).

### Outdoor learning environment practices

The outdoor learning environment practices are shown in Table 3. Most respondents (> 75%) reported providing outdoor active free play two or more times per day. Almost all (98.32%) reported at least one NLI indicator was present at their ECE, with an average of 5.7 of 12 NLI (SD = 2.55, range = 0, 12) indicators present. One third of centers reported less than four indicators with 75% of centers reporting three to eight indicators. Only one center reported all 12 best practices indicators. The most commonly reported indicators are providing sufficient support of gross motor activities, open, grassy areas, and play equipment (> 70%), and having natural, loose materials, and shade structures (> 65%). Areas for improvement include having sufficient trees (51%), having looping pathways, outdoor classroom/program base/storage, at least 10 play and learning settings (< 40%), a designated vegetable garden (< 20%), sufficient shrubs, and edible fruit or nut trees (< 10%).

**Table 3:** Reported Practices Regarding Outdoor Learning Environments in Participating Texas Child Care Centers^a^

	%	(*n*)
Outdoor active free play is provided to all toddlers & preschoolers^c^		
< 1x per wk.	0.21	(1)
4x per wk	5.47	(26)
1x per day	15.79	(75)
**≥ 2x per day**	**78.53**	**(373)**
Outdoor learning environment & activities are linked to enforce learning^d^		
Never	1.27	(6)
Rarely	6.77	(32)
Sometimes	50.32	(238)
**Often**	**41.65**	**(197)**
Best Practice Indicators for a Model Outdoor Learning Environment^be^		
Total indictors reported (M, SD)	5.74	(2.55)
	Indicator Reported	
	Yes	
	%	(*n*)
PA is supported by OLE (running, jumping on/off, crawling through, rolling, swinging, throwing, balancing, climbing)	87.27	(418)
Open, grassy area for games & events	74.11	(355)
Wheeled toys, portable play equipment, & play materials available	73.70	(353)
Natural, loose materials (leaves, sticks, gravel, seeds) available for play	69.52	(333)
Sufficient man-made shade structures	66.81	(320)
Trees provide cover for about 1/3rd of outdoor area	51.15	(245)
Looping, curvy primary pathways for circulation and wheeled toys	41.34	(198)
≥ 10 outdoor play & learning settings for activities	38.00	(182)
Outdoor classroom/program base/storage available for tools, equipment & materials for outdoor learning	37.79	(181)
Designated vegetable garden	17.54	(84)
Shrubs (3 for every 100 sq. ft), including ≥1/4 fruiting shrubs & vines	8.98	(43)
≥ 1/4 of trees are edible fruit or nut species	7.52	(36)

NOTE: Boldface indicates best practice recommendation

^a^ Expected *N* = 481. Actual *n* = 479, due to missing data

^b^ Outdoor learning environment best practice indicator questions were check all that apply option. Answers were dichotomized (Reported/Not reported)

^c^ Early Childhood PA Survey Question based on TX CORD

^d^ Early Childhood PA Survey Question newly created based on Texas Rising Star standard

^e^ Early Childhood PA Survey Question newly created based on National Learning Initiative

Abbreviations: x, time; wk., week; M, mean; SD, standard deviation; PA, physical activity; OLE, outdoor learning environment; sq., square; ft., feet

### Physical activity and screen time policies

The reported policies related to physical activity and screen time are shown in Table 4. More than 80% of centers have a least one policy related to physical activity or screen time, with an average of 3.92 physical activity policies (SD = 3.00, range = 0, 10) and an average of 1.44 screen time policies (SD = 1.65, range = 0, 6). Policies regarding amount of time and clothing that allows children and teachers/caregivers to be physically active was the highest reported (> 65%). Clothing policies included information about shoes (e.g. closed-toed, non-sandal type shoes) and clothes which allow for movement and play indoors and outdoors, such as properly fitting clothes (too loose fit can get caught on equipment or too tight which can restrict abilities). Policy areas endorsed by less than 50% of respondents include type of activity provided (structured or unstructured) (< 50%), education for teachers, children, and families regarding physical activity (< 35%), and limiting seating time (< 30%). Reported policies regarding screen time were less common (< 46%). Areas for improvement included not using screen time as a reward, providing professional development for teachers and staff, and providing education for families on screen time (< 15%).

**Table 4:** Reported Physical Activity and Screen Time Policies in Participating Texas Child Care Centers^ab^

	Policy reported	
	Yes	
	%	(*n*)
The facility has a written policy on PA and/or screen time	81.70	(393)
Physical Activity^cd^		
Total PA policies reported (M, SD)	3.92	(3.00)
Shoes & clothes that allow children and teachers/caregivers to actively participate in PA	66.45	(303)
Amount of time provided each day for indoor & outdoor PA	62.50	(285)
Unstructured (active free play) PA play	48.25	(220)
Not withholding PA as punishment	40.57	(185)
Supporting PA (e.g. staff involved during active play time, visible display in classrooms & common areas)	39.91	(182)
Structured (adult-led active play) PA play	35.53	(162)
Education for teachers/caregivers on children’s PA	33.33	(152)
Limiting long periods of seated time for children	28.07	(128)
Education for children on PA	25.22	(115)
Education for families on children’s PA	12.28	(56)
Screen Time^c^		
Total screen time policies reported (M, SD)	1.44	(1.65)
Amount of screen time allowed	45.37	(206)
Types of programming allowed	34.14	(155)
Appropriate supervision & use of screen time in classrooms	30.84	(140)
Not using screen time as a reward or to manage challenging behaviors	14.54	(66)
Professional development on screen time	12.56	(57)
Education for families on screen time	6.61	(30)

^a^Policy questions were check all that apply option. Answers were dichotomized (Reported/Not reported)

^b^Total N = 481, n varies from 453 to 456 due to missing data

^c^Early Childhood PA Survey Question based on NAP SACC

^d^Early Childhood PA Survey Question based on TX CORD

Abbreviations: PA, physical activity; M, mean; SD, standard deviation

## Discussion

We examined the extent to which non-Head Start ECE centers in Texas report meeting best practice recommendations and written policies for physical activity and screen time behaviors as well as aspects of the outdoor learning environment. While many centers reported meeting best practices for screen time and physical activity, there is still room for improvement for some best practices and center-level policies. With 1.5 million children under the age of four [27] and up to 950,000 children in Texas enrolled in center-based care [14], child care centers have the potential for a substantial public health impact to improve the health behaviors of preschool-aged children.

While most Texas centers reported meeting best practice recommendations for screen time activities, there were areas for improvement in regards to meeting physical activity best practices. Physical activity practices that were met less than 35% of the time and could be improved included never placing infants in seats, time provided for indoor and outdoor play, having teachers join children in active play, the facility showing support for physical activity (e.g. posters, pictures, or books about physical activity displayed in rooms), and providing professional development. Providing time for outdoor play is especially important as physical activity levels in young children are higher during outdoor time than during indoor activities [28] and outdoor time has been found to be the strongest predictor of meeting physical activity best practices [29]. In addition, most centers had outdoor learning environments that did not meet best practice design recommendations as centers reported, on average, less than half of the 12 outdoor learning best practice indicators, with one third of centers reported less than four outdoor learning indicators. Improving outdoor settings by creating environments with nature, vegetation, pathways, and play and learning settings may increase child physical activity levels [9–11].

Written policies need to include providing education to teachers, children, and their families to support healthy behaviors in ECE settings. While a large percentage of participants self-reported having written policies regarding physical activity, the lack meeting best practice recommendations suggests there needs to be an increased focus on implementation within the ECE. Current recommendations in Texas to improve the outdoor learning environments include Texas Rising Star, a voluntary quality-based child care rating system based on the State’s Minimum Child Care Licensing Standards, whose requirements include measures regarding outdoor learning environments [30]. Only one center in this study reported meeting all 12 of NLI best practice indicators and one-third reported less than four indicators were present at their center. Thus, there is a need to work with centers to increase their knowledge of best practice indicators for the outdoor environment to help centers improve their social and physical environment.

In comparison with other statewide surveys previously conducted in Washington [29], Minnesota and Wisconsin [31], and Oklahoma [32], ECE programs in Texas report having written policies and staff professional development regarding physical activity more frequently than other states. Texas ECE centers that completed the survey are also more likely to self-report meeting best practice recommendations for amount of physical activity provided than those in Washington, Minnesota, and Wisconsin. The respondents in Texas reported meeting less physical activity best practices than the study in Oklahoma, and the authors of the Oklahoma study noted differences between meeting best practice recommendations by accreditation status [32]. Accreditation status may be an important moderator in Texas as well and should be examined in future research. To improve the number of centers meeting best practices, it may be beneficial to examine efforts occurring in states where a majority of centers are meeting best practices and implementing policies. Of note in Texas, the DSHS has efforts underway to increase children’s access to physical activity in the child care environment through a statewide initiative to increase awareness and improvement within the ECE outdoor built environment. Moreover, the Texas the Early Childhood Health and Nutrition Interagency Council, created by Texas Senate Bill 395 to improve the health of young children in Texas, is currently preparing a report of efforts from state agencies and stakeholders, in order to make recommendations to the Texas legislature and Governor on ways to improve the health of Texas children through physical activity.

### Limitations

This study has limitations. The findings are from a convenience sample and thus do not represent all child care facilities in Texas, limiting generalizability. The use of incentives, seen in other statewide surveys [29, 31, 32], may have been one way to increase participation rate and overall generalizability; however, the resources were not available for this study. Additionally, the self-reported nature of the survey may introduce a response bias. Social-desirability bias may have resulted in the respondents underestimating the percentage of undesirable practices and/or overestimating the percentage of desirable practices or policies. However, this bias can be assumed across previous statewide studies and should not affect Texas disproportionately. Finally, this study did not collect sociodemographic information of the children served, thus we are unable to examine potential center differences at the broader regional level. Future research should examine if differences in policies and practices exist across center and geographic characteristics, which could inform stakeholders and policy makers. The strengths of this study include the use of widely used scales (NAP SACC and Yale Rudd Center Child Care Nutrition and Physical Activity Assessment) to assess physical activity practices, which enables comparison to other studies. Additionally, this is one of the first statewide surveys to include measures of outdoor learning environments.

### Conclusions

The study was the first statewide survey of outdoor environments, physical activity and screen time-related practices and policies in ECE centers in Texas. The survey results show there is room for improvement in meeting best practices in ECE centers, specifically within physical activity and outdoor learning environments. Although there are state regulations in Texas requiring specific minutes of moderate to vigorous physical activity for students enrolled in prekindergarten within school districts [33], there are currently no separate provisions for child care. Revising licensing standards to include information regarding specific minutes of physical activity provided, providing professional training for child care centers in physical activity and screen time practice, and dedicating resources to help centers enact and modify written policies and implement programs could promote physical activity and reduce sedentary time of children attending ECE centers. Additionally, including outdoor learning environments in licensing standards and dedicating resources to help centers improve their outdoor learning environments could promote increased activity. Future statewide work should continue to use widely validated surveys in order for comparison across states, however researchers should consider including device-based measures in addition to self-report methods. Future research should continue to assess written policies within ECE centers, help facilities modify existing written policies, and develop programs that will help centers implement written policies that promote healthy behaviors.

## Additional file

Additional file 1: Title of data: Early Childhood Physical Activity Survey. Description of data: This is the full Early Childhood Physical Activity Survey which was developed using questions from the Nutrition and Physical Activity Self-Assessment for Child Care assessment tool (NAP SACC), Yale Rudd Center Child Care Nutrition and Physical Activity Assessment, Texas Childhood Obesity Research Demonstration (CORD) child care survey, the Natural Learning Initiative’s (NLI) Best Practice Indicators for a Model Outdoor Learning Environment Toolkit and other items developed specifically for this study. (PDF 165 kb)

## Abbreviations

CORD: Texas Childhood Obesity Research Demonstration
DSHS: Texas Department of State Health Services
ECE: Early Care and Education
HPCDPS: Health Promotion and Chronic Disease Prevention Section
NAP SACC: Nutrition and Physical Activity Self-Assessment for Child Care
NLI: Natural Learning Initiative
POD: Preventing Obesity by Design
SD: Standard deviation
