# Supplementary material for: Physical activity, screen time, and outdoor learning environment practices and policy implementation: a cross sectional study of Texas child care centers
Source: BMC Public Health. 2019 Mar 7;19:274. doi: 10.1186/s12889-019-6588-5 (PMC6407214; doi:10.1186/s12889-019-6588-5)
Supplement: Supplementary file 1 — Title of data: Early Childhood Physical Activity Survey. Description of data: This is the full Early Childhood Physical Activity Survey which was developed using questions from the Nutrition and Physical Activity Self-Assessment for Child Care assessment tool (NAP SACC), Yale Rudd Center Child Care Nutrition and Physical Activity Assessment, Texas Childhood Obesity Research Demonstration (CORD) child care survey, the Natural Learning Initiative’s (NLI) Best Practice Indicators for a Model Outdoor Learning Environment Toolkit and other items developed specifically for this study. (PDF 165 kb) [file 12889_2019_6588_MOESM1_ESM.pdf]

Early Childhood Physical Activity Survey
Early Childhood Health and Nutrition Interagency Council

The survey should be completed by a person responsible for overseeing the physical activity (indoor and outdoor play) of the children in care (e.g., day care home provider, center teacher, center director, or administrator).

For this survey, use the age groups below when answering questions:
Infants (0 – 12 months)
Toddlers (13 – 23 months)
Preschool (age 2 – 5 years)
All Children (Toddlers and Preschool)

* 1. What is today's date?

	MM	DD	YYYY
Today's Date	☐ /	☐ /	☐

* 2. What is your current position? Check all that apply.

- ☐ Principal
- ☐ Center Director
- ☐ Center Teacher
- ☐ Child Care Home Provider
- ☐ Other (please specify)

* 3. Do you work at a:

- ☐ Child care center
- ☐ Child care home
- ☐ Early Head Start
- ☐ Head Start
- ☐ State-funded Pre-K program

4. How many infants (age 0-12 months) are currently enrolled in your facility?

5. How many toddlers (13-23 months) are currently enrolled in your facility?

6. How many preschool age children (age 2-5 years) are currently enrolled in your facility?

7. How many children age 6 and up are currently enrolled in your facility?

* 8. How long have you worked at this facility?

- Less than 1 year
- 1 year
- 2-5 years
- 6-10 years
- More than 10 years

* 9. Does your facility participate in programs that address physical activity? Choose all that apply.

- Texas Rising Star Program
- National Association for the Education of the Young Child (NAEYC)
- National Early Childhood Program Accreditation (NECPA)
- National Accreditation Commission (NAC) for Early Care and Education Programs
- National Association of Family Child Care (NAFCC)
- Let's Move Child Care
- I Am Moving, I Am Learning
- Coordinated Approach to Child Health (CATCH)
- None of the above

Other (please specify)

Answer each question as best you can. If none of the answer choices seem quite right, just pick the closest fit. Answer choices in parentheses ( ) are for half-day programs. Full-day programs should use the answer choices without parentheses.

10. The amount of time provided to preschool children (age: 2 – 5 years) for indoor and outdoor physical activity each day is:

Definition: Physical activity is any movement of the body that increases heart rate and breathing above what it would be if a child was sitting or resting. Examples include: walking, running, crawling, climbing, jumping and dancing.

- ◯ Less than 60 minutes (Half-day: less than 30 minutes)
- ◯ 60 – 89 minutes (Half-day: 30 – 44 minutes)
- ◯ 90 – 119 minutes (Half-day: 45 – 59 minutes)
- ◯ 120 minutes or more (Half-day: 60 minutes or more)

11. The amount of adult-led (structured) physical activity provided to preschool children (age: 2 – 5 years) is:

Definition: Adult-led activities and lessons can be led by teachers or outside presenters. Examples include dancing, music and movement, motor development lessons, physically active games, and tumbling. The total amount of adult-led activity time may include multiple short activities added up over the course of the day.

- ◯ Less than 30 minutes (Half-day: less than 10 minutes)
- ◯ 30-44 minutes (Half-day: 10 – 19 minutes)
- ◯ 45 – 59 minutes (Half-day: 20 – 29 minutes)
- ◯ 60 minutes or more (Half-day: 30 minutes or more)

12. The amount of time provided to toddlers (age 13-23 months) for indoor and outdoor physical activity each day is:

- Less than 60 minutes (Half-day: less than 15 minutes)
- 60 – 74 minutes (Half-day: 15 – 29 minutes)
- 75 – 89 minutes (Half-day: 30 –44 minutes)
- 90 minutes or more (Half-day: 45 minutes or more)

13. During unstructured physical activity playtime, teachers or caregivers:

Definition: Unstructured physical activity is the active free play that children do when they are free to play on their own. Free play can include swinging, sliding, climbing pushing, pulling, riding, or playing chase. In free play, the adult watches and encourages active play but does not lead the children’s play.

- Rarely or never join children in active play (mostly sit or stand)
- Sometimes join children in active play
- Often or always join children in active play
- Often or always join children in active play and make positive statements about the activity

14. Our program offers “tummy time” to non-crawling infants (age 0-12 months):

Definition: Tummy time is supervised time when an infant is awake and alert, lying on her/his belly. Opportunities for tummy time should last as long as possible to help infants learn to enjoy it and build their strength. For infants who are not used to it or do not enjoy it, each period of tummy time can start at 1 – 2 minutes, and build up to 5-10 minutes over time.

- 1 time per day or less (Half-day: 1 time every other day)
- 2 times per day (Half-day: 1 time per day)
- 3 times per day (Half-day: 2 times per day)
- 4 times per day or more (Half-day: more than 2 times per day)

15. Teachers offer developmentally appropriate portable play equipment to infants during tummy time and other indoor activities:

Definition: Portable play equipment for infants includes balls, soft blocks, mirrors to view self and rattles.

- Rarely or never
- Sometimes
- Often
- Always

16. Outside of nap and meal times, the longest that infants (age 0-12 months) spend in seats, swings, or ExcerSaucers at any one time is:

- ◯ 30 minutes or more
- ◯ 15-29 minutes
- ◯ 1-14 minutes
- ◯ Infants are never placed in seats, swings, or ExcerSaucers

17. Outside of nap and meal times, the longest that preschool children (age: 2 – 5 years) and toddlers (13 to 23 months) are expected to remain seated at any one time is:

- ◯ 30 minutes or more
- ◯ 20-29 minutes
- ◯ 15-19 minutes
- ◯ Less than 15 minutes

18. For children 2 years of age and older, the amount of screen time allowed in our program each week is:

Definition: screen time includes any time spent watching shows or playing games (including active video games) on a screen. Screens can include televisions; desktop, laptop, or tablet computers; or smart phones.

Minutes of educational screen time per week ____________________

Minutes of recreational screen time per week ____________________

19. For children under 2 years of age, the amount of screen time allowed in our program each week is:

Minutes of educational screen time per week ____________________

Minutes of recreational screen time per week ____________________

20. Staff members restrict active play time for children who misbehave:

- ◯ Never
- ◯ Some staff members
- ◯ Most staff members
- ◯ All staff members

21. Outdoor active free play is provided for all children (toddlers and preschool):

- ◯ 1 time per week or less
- ◯ 4 times per week
- ◯ 1 time per day
- ◯ 2 or more times per day

22. Outdoor learning environment and activities are linked to enforce indoor learning:

- ◯ Never
- ◯ Rarely
- ◯ Sometimes
- ◯ Often

23. Which of the following best practice indicators for a model outdoor learning environment does your facility include? Choose all that apply.

- ☐ There are at least 10 outdoor play and learning settings for different activities.
- ☐ There are looping, curvy primary pathways provided for circulation and available for children to use with wheeled toys.
- ☐ There is an open, grassy area for games and events for children.
- ☐ There are sufficient (man-made) shade structures, in addition to trees, to provide children with protection from the sunlight.
- ☐ There are sufficient different types of natural, loose materials (such as leaves, sticks, gravel, seeds) present and children are allowed to play freely with them.
- ☐ There are sufficient different types of wheeled toys, portable play equipment (such as balls, blocks, jump rope), and play materials (such as dress-up clothes) available to stimulate creative play and children are allowed to play freely with them.
- ☐ There are sufficient types of physical activities, including gross motor activities supported by the outdoor learning environment (such as running, jumping on/off, crawling through, rolling, swinging, throwing, balancing, climbing).
- ☐ There are sufficient trees providing cover for about 1/3rd of the outdoor area.
- ☐ At least ¼ of trees are edible fruit or nut species.
- ☐ There are sufficient shrubs (about 3 for every 100 sq. ft.), including at least ¼ fruiting shrubs and vines.
- ☐ There is a designated vegetable garden with sufficient produce for repeated opportunities for snacking and/or meals during growing seasons.
- ☐ There is an outdoor classroom/ program base/storage available for tools, equipment and materials for outdoor learning.
- ☐ None of the above

24. Physical activity education for children (motor-skill development) is provided through a standardized curriculum:

Definition: Standardized curriculum is the academic content of a child's day. Curriculum is an imperative, complex concept that includes a host of activities and learning experiences, that include daily activities -- circle time, song time, active play time, story time, craft time, etc. Standardized curriculums include but not limited to: Bank Street, Reggio Emilia, and High/Scope.

- Never
- 1 time per month
- 2-3 times per month
- 1 or more time per week

25. The facility shows visible support for physical activity by:

- No posters, pictures, or books about physical activity are displayed.
- A few posters, pictures, or books about physical activity displayed in a few rooms.
- Posters, pictures, or books about physical activity are displayed in most rooms.
- Posters, pictures, or books about physical activity are displayed in all rooms.

* 26. Does your facility have a written policy on physical activity and/or screen time?

Definition: A written policy can include any written guidelines about your program's operations or expectations for teachers, staff, children, and families. Policies can be included in parent handbooks, staff manuals and other documents.

- Yes
- No

27. Which of the following topics are addressed by the written policy? Choose all that apply.

- [ ] No written physical activity policy
- [ ] Structured (adult-led active play) physical active play
- [ ] Unstructured (active free play) physical active play
- [ ] Amount of time provided each day for indoor and outdoor physical activity
- [ ] Limiting long periods of seated time for children
- [ ] Not withholding physical activity as punishment
- [ ] Shoes and clothes that allow children and teachers/caregivers to actively participate in physical activity
- [ ] Education for teachers/caregivers on children's physical activity
- [ ] Education for children on physical activity
- [ ] Education for families on children's physical activity
- [ ] Supporting physical activity (e.g. staff involved during active play time, visible display in classrooms and common areas)
- [ ] Policy does not include these topics
- [ ] Other (please specify)

28. Our written policy on screen time includes the following topics: Choose all that apply.

- [ ] No written screen time policy
- [ ] Amount of screen time allowed
- [ ] Types of programming allowed
- [ ] Appropriate supervision and use of screen time in classrooms
- [ ] Not using screen time as a reward or to manage challenging behaviors
- [ ] Professional development on screen time
- [ ] Education for families on screen time
- [ ] Policy does not include these topics
- [ ] Other (please specify)

29. Which of the following prevent you from promoting physical activity in your facility? Choose all that apply.

- [ ] Lack of support from administration
- [ ] Lack of support from teachers
- [ ] Lack of support from parents/families
- [ ] Lack of staff training and education in the area of physical activity
- [ ] Lack of space or equipment
- [ ] Lack of established policies on physical activity (e.g. curriculum, materials)
- [ ] Insufficient funds
- [ ] Lack of physical education resources
- [ ] Other (please specify)

* 30. Teachers and staff receive professional development on children’s physical activity:

Definition: For this assessment, professional development on children’s physical activity does not include training on playground safety. Professional development can include taking in-person or online training for contact hours or continuing education credits. It can also include information presented at staff meetings.

- ○ Never
- ○ Less than 1 time per year
- ○ 1 time per year
- ○ 2 times per year or more

31. Professional development for current staff on children's physical activity has included the following topics: (Choose all that apply)

- [ ] Recommended amounts of daily physical activity for young children
- [ ] Encouraging children's physical activity
- [ ] Limiting long periods of seated time for children
- [ ] Children's motor skill development
- [ ] Communicating with families about encouraging children's physical activity
- [ ] Our program's policies on physical activity
- [ ] Other (please specify)

Early Childhood Physical Activity Survey
Early Childhood Health and Nutrition Interagency Council

Demographics

32. What is your age?

33. What is your gender?

* 34. What is your facility's zip code?

* 35. What language(s) do the child care providers primarily speak in your center, home or facility?

- Only English
- More English than another language
- Only Spanish
- More Spanish than another language
- Both English and Spanish
- Language other than English or Spanish (please specify)

36. What is the highest grade or year of school you completed?

- Never attended school or only attended kindergarten
- Elementary (grades 1 – 8)
- Some high school (grades 9 – 12)
- High School graduate or GED
- Child Development Associate (CDA)
- Some college or technical school (College 1 year to 3 years)
- College graduate (College 4 years or more)
- Graduate Degree

37. Are you Hispanic or Latino?

- ◯ Yes
- ◯ No

38. Which one of these groups would you say best represents your race?

- ◯ White
- ◯ Black or African American
- ◯ Asian
- ◯ Native Hawaiian or Other Pacific Islander
- ◯ American Indian or Alaska Native
- ◯ More than one race
- ◯ Other (please specify)
